# Persistent Polyuria in Severe Hyponatremia Secondary to Beer Potomania and Hypokalemia: A Case Report

**DOI:** 10.7759/cureus.85503

**Published:** 2025-06-07

**Authors:** Rimi Tanii, Noboru Tahara, Koichi Hayashi, Shigeki Fujitani

**Affiliations:** 1 Emergency and Critical Care Medicine, St. Marianna University School of Medicine, Kawasaki, JPN

**Keywords:** beer potomania, hypokalemia, hyponatremia, hypotonic polyuria, vasopressin

## Abstract

Multiple electrolyte disorders often coexist in patients with beer potomania, including hypotonic hyponatremia caused by excessive beer consumption and poor solute intake. While hyponatremia is well recognized, its management can be complicated by concurrent hypokalemia and hypomagnesemia, and the impact of other electrolyte imbalances on its treatment remains unclarified. Here, we present a case of severe hyponatremia accompanied by persistent polyuria as a consequence of severe hypokalemia and hypomagnesemia. A 46-year-old man with a history of heavy beer consumption was admitted to the hospital with impaired mental status. Laboratory findings revealed multiple electrolyte disorders, including hyponatremia (111 mmol/L), hypokalemia (1.8 mmol/L), and hypomagnesemia (1.4 mg/dL). Initial treatment with normal saline caused massive diuresis (3,500 mL in six hours) and a rapid rise in serum sodium (119 mmol/L). To prevent overcorrection and subsequent osmotic demyelination, 5% dextrose and vasopressin were administered; however, it failed to adequately control diuresis. As severe hypokalemia and hypomagnesemia were corrected, the antidiuretic response of vasopressin was restored, leading to an improvement in urine output. Vasopressin was tapered and discontinued after serum sodium reached 132 mmol/L on hospital day six. In conclusion, this case highlights the importance of individualized fluid and electrolyte management in beer potomania, especially when antidiuretic hormone responsiveness is impaired, possibly due to hypokalemia and hypomagnesemia.

## Introduction

Chronic alcoholism is associated with a wide range of metabolic complications, including multiple electrolyte disorders as well as nutritional problems [1]. Among alcoholic beverages, beer contains only trace amounts of electrolytes and solutes. When consumed in large quantities over a relatively short period, particularly with insufficient dietary intake, it can cause a distinct clinical syndrome known as beer potomania [2]. This condition is characterized by various electrolyte disorders, including severe hyponatremia, hypokalemia, hypophosphatemia, and hypomagnesemia [1,2]. A previous review has reported that such electrolyte disturbances are frequently observed in patients with chronic alcohol-use disorder, with hyponatremia present in approximately 17% of the cases [1]. Among these, beer potomania represents a specific mechanism of hypotonic hyponatremia caused by solute depletion of poor nutritional intake rather than inappropriate antidiuretic hormone (ADH) secretion [2,3]. This syndrome remains underrecognized and is likely underestimated in clinical practice. A review of 22 published cases found that 18% of patients with beer potomania developed osmotic demyelination syndrome (ODS) [4]. Unlike other alcohol-related hyponatremic syndromes, such as cirrhosis-associated syndrome of inappropriate secretion of antidiuretic hormone (SIADH), beer potomania involves a unique combination of low solute intake and free water retention, leading to impaired urinary dilution capacity and profound hypotonic hyponatremia [5]. Whereas SIADH is characterized by inappropriately elevated ADH secretion that persists despite hypoosmolality, beer potomania typically features suppressed ADH secretion with impaired water clearance due to low solute load, reflecting a fundamentally different mechanism of hyponatremia [6]. This is crucial to understand because the therapeutic approach differs substantially. Furthermore, the correction of these disturbances is not always straightforward, but the impact of one electrolyte disorder on others may complicate the treatment strategies [7].

Here, we describe a patient with beer potomania who presented with multiple electrolyte disorders, accompanied by persistent polyuria. Initial treatment with saline improved serum sodium but led to massive diuresis. To prevent overcorrection and serious complications such as ODS, 5% dextrose and vasopressin were administered. Although vasopressin was initially ineffective, gradual correction of hypokalemia and hypomagnesemia improved the renal response to vasopressin. Attempts to discontinue vasopressin resulted in recurrent polyuria, but successful cessation was achieved when the serum sodium level reached 132 mmol/L. This case highlights the importance of early recognition and individualized management of beer potomania, particularly in patients at high risk of ODS.

This article was previously presented as a meeting abstract at the 52nd Annual Academic Meeting of the Japanese Society for Acute Medicine on October 15th, 2024.

## Case presentation

A 46-year-old man with a history of chronic alcoholism and alcoholic liver cirrhosis presented to the emergency room (ER) with impaired mental status. He had a past medical history of asthma in childhood. He had not eaten much for the past months but consumed an excess amount of beer (more than 4 L/day). He did not take any medications, including diuretics. On presentation, his vital signs included an oxygen saturation of 90% with ambient air, and others were within normal limits. He was agitated and disoriented, with a Glasgow Coma Scale (GCS) score of 9 (E3V1M5). The physical examination showed dry skin, no focal neurological deficits, and no peripheral edema.

**Figure 1:**
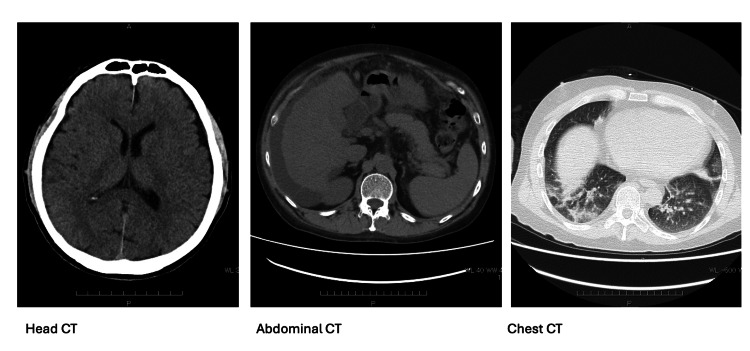
CT scan of the head, abdominal, and chest (axial views) on admission. Head CT (axial view) shows no acute intracranial changes, and abdominal CT (axial view) demonstrates hepatic cirrhosis with mild ascites, consistent with underlying chronic alcohol use. Chest CT (axial view) demonstrates pneumonia.

His laboratory findings revealed multiple derangements in serum electrolytes (sodium, 111 mmol/L; potassium, 1.8 mmol/L; chloride, <80 mmol/L; magnesium, 1.4 mg/dL; phosphate, 2.0 mg/dL) and a marked increase in serum bicarbonate (45.2 mmol/L; Table 1). His serum and urine osmolalities were 225 mOsm/kg and 143 mOsm/kg, respectively, and his urine sodium concentration was less than 20 mmol/L. The urine sample was obtained after initial administration of saline. Other laboratory data showed a normal serum creatinine level (0.56 mg/dL) but low blood urea nitrogen (BUN, 3.6 mg/dL) and impaired liver functions (total bilirubin, 14 mg/dL; aspartate aminotransferase, 289 IU/L; alanine aminotransferase, 49 IU/L; albumin, 2.7 g/dL). Thyroid and cortisol levels were normal. CT scan of the brain was negative for acute changes, but abdominal CT showed cirrhosis with a mild level of ascites (Figure 1). Chest CT demonstrated pneumonia, for which he initiated 4 L/min of oxygen and intravenous ceftriaxone.

**Table 1:** Initial laboratory data in the emergency department. Initial biochemical findings show several electrolyte disturbances, including severe hyponatremia, hypokalemia, and hypomagnesemia. Elevated bicarbonate (45.2 mmol/L) and metabolic alkalosis also exist, accompanied by liver dysfunctions (elevated AST and low albumin). WBC, white blood cell count; RBC, red blood cell count; Na, sodium; K, potassium; Cl, chloride; Ca, calcium; iP, inorganic phosphate; Mg, magnesium; pCO₂, partial pressure of carbon dioxide; pO₂, partial pressure of oxygen; BUN, blood urea nitrogen; Alb, albumin; AST, aspartate aminotransferase; ALT, alanine aminotransferase; ALP, alkaline phosphatase; TSH, thyroid-stimulating hormone; T4, thyroxine; ACTH, adrenocorticotropic hormone; Cre, creatinine; Osm, osmolality.

Test	Results	References
Blood		
WBC (x10^3^/µL)	3.5	3.3-8.6
RBC (x10^6^/µL)	3.62	4.35-5.55
Hemoglobin (g/dL)	12.9	13.7-16.8
Platelets (x10^4^/µL)	4.8	15.8-34.8
Na (mmol/L)	111	138-145
K (mmol/L)	1.8	3.6-4.8
Cl (mmol/L)	<80	101-108
Ca (mg/dL)	7.5	8.8-10.1
iP (mg/dL)	2.0	2.7-4.6
Mg (mg/dL)	1.4	1.8-2.4
pH	7.547	
pCO_2_ (mmHg)	52.0	
pO_2_ (mmHg)	30.5	
Bicarbonate (mmol/L)	45.2	
Glucose (mg/dL)	135	73-109
BUN (mg/dL)	3.6	8.0-20.0
Creatinine (mg/dL)	0.56	0.65-1.07
Serum Alb (g/dL)	2.7	4.1-5.1
AST (IU/L)	289	13-30
ALT (IU/L)	49	10-42
ALP (IU/L)	260	38-113
Total bilirubin (mg/dL)	14	0.4-1.5
Thyroid hormone: free T4 (pg/mL)	1.28	0.9-1.7
TSH (µIU/mL)	2.39	0.61-4.23
Cortisol (μg/dL)	11.8	7.07-19.6
ACTH (pg/mL)	42.4	7.2-63.3
Serum osmolality (mOsm/kg)	225	270-295
Urine		
Na (mmol/L)	<20	
K (mmol/L)	11.3	
Cl (mmol/L)	<50	
Cre (mg/dL)	45.6	
Urine osmolality (mOsm/kg)	143	

**Figure 2:**
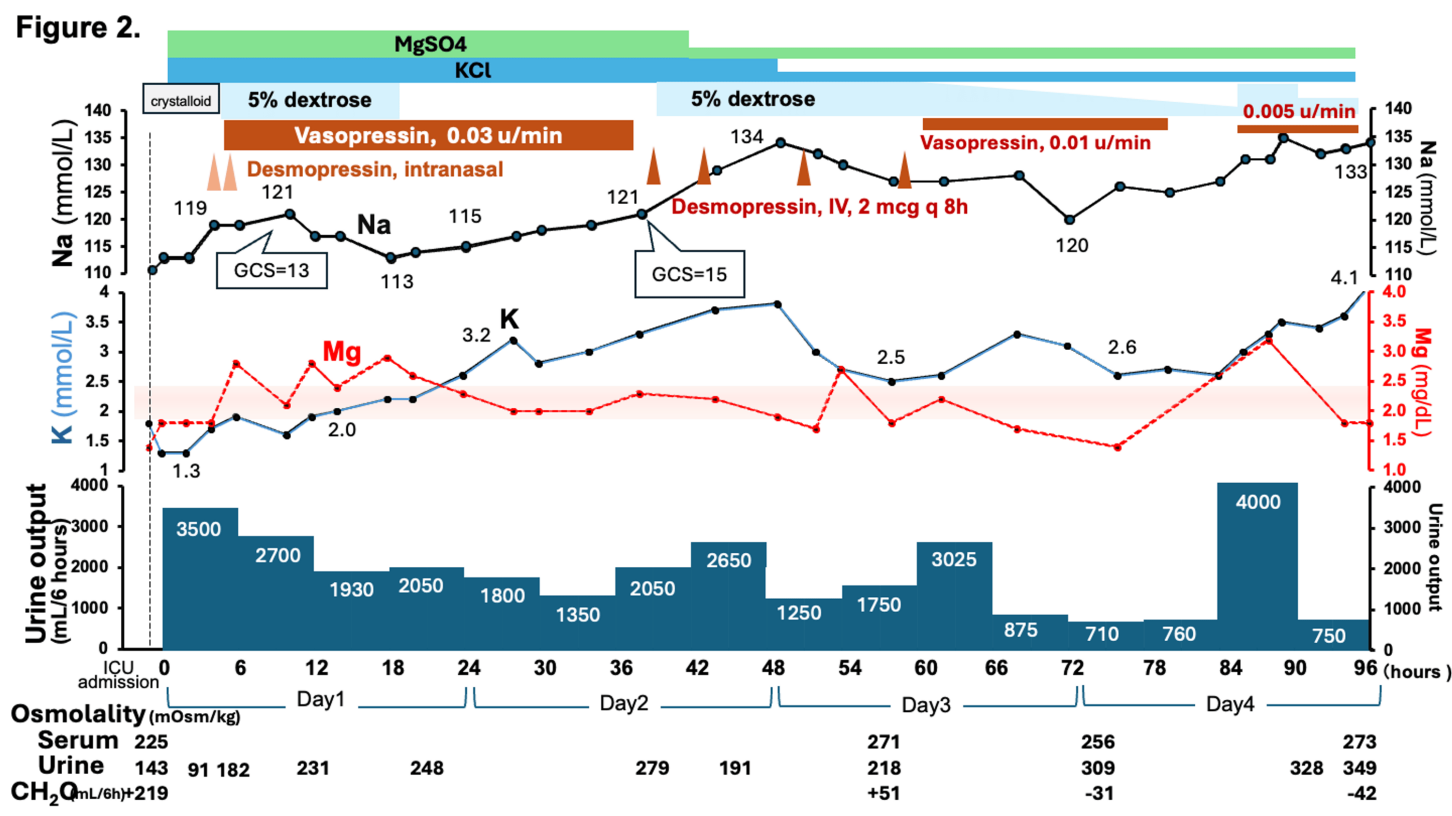
Temporal course of serum electrolytes and urine output. Trends in serum sodium (Na), potassium (K), and magnesium (Mg) are plotted alongside six-hour urine output. Intravenous (IV) fluid administration (balanced crystalloid and 5% dextrose) and vasopressin/desmopressin treatment are also indicated. A transient increase in urine output and serum sodium was observed shortly after ICU admission. Free water clearance (CH₂O) was calculated at selected time points and showed a transition from marked water diuresis to an antidiuretic state. It was strongly positive (+219 mL/6 hours upon ICU admission) to mildly positive (+51 mL/6 hours at 56 hours), and later became negative (–31 mL/6 hours at 72 hours, –42 mL/6 hours at 96 hours), indicating an improvement in renal responsiveness to vasopressin. * CH₂O represents the net volume of solute-free water excreted by the kidneys, with positive values indicating water diuresis.

In the ER, he received a 1-liter bolus of normal saline intravenously, followed by continuous infusion at 500 mL/h for approximately two hours. Upon ICU admission, the intravenous fluid was switched to a balanced crystalloid at 80 mL/h. Serum sodium and other electrolytes were monitored every two hours, and potassium, magnesium, and phosphate were supplemented as necessary. Vitamin B1 was also administered. Subsequently, he exhibited hypotonic polyuria (91 mOsm/kg, 3 L/4 h) alongside a reduction in serum potassium (1.3 mmol/L) and a rapid rise in serum sodium to 119 mmol/L within six hours (Figure 2). To avoid the risk of overcorrection, the infusion of crystalloid was discontinued and replaced with 5% dextrose at a rate of 600 ml/h. Desmopressin was also administered intranasally, and vasopressin was initiated intravenously at a rate of 0.03 units/min. Despite these interventions, massive polyuria continued (2.7 L/6 h), and he required large amounts of potassium supplementation (520 mmol of potassium chloride on the first day). In parallel with the gradual increase in serum potassium to levels higher than 2.0 mmol/L, alongside the increase in magnesium, the amount of urine decreased, and the serum sodium level was elevated. Ten hours after ICU admission, when the sodium level reached 121 mmol/L, his GCS score improved to 13 (E3V4M6).

On hospital day two, with continued administration of a large amount of potassium (620 mmol), the serum potassium reached 3.2 mmol/L, and urine output decreased. At 38 hours after ICU admission, his GCS improved further to 15 (E4V5M6) when the sodium level was 121 mmol/L. Hence, the vasopressin infusion was discontinued and replaced with intermittent intravenous desmopressin (2 mcg every eight hours). However, 5% dextrose was resumed due to the recurrent polyuria and a rapid rise in serum sodium concentration (19 mmol/L in 24 hours). On hospital day three (65 hours after ICU admission), due to persistent polyuria, vasopressin infusion was resumed at 0.01 unit/min. On day four, when the serum sodium level reached 125 mmol/L, a second attempt at discontinuing vasopressin infusion resulted in the recurrence of polyuria; vasopressin was promptly reinitiated at 0.005 units/min. Hence, vasopressin was gradually tapered and completely stopped on day six, whilst no overcorrection or polyuria ensued. The patient was transferred to the general ward without any neurological abnormalities on hospital day seven.

## Discussion

Heavy beer consumption is associated with poor solute intake and promotes the development of severe hyponatremia and various electrolyte disorders, including hypokalemia, hypomagnesemia, and hypophosphatemia [1]. In beer potomania, a large volume of hypotonic fluid in the context of poor dietary intake leads to reduced renal osmole excretion, which limits free water clearance to approximately 3-4 L/day, contributing to the development of hyponatremia [2,3]. This mechanism underlies the pathogenesis of hyponatremia in our case, in which the patient consumed an excess amount of beer (more than 4 L/day) and was malnourished, as shown by a low level of BUN (3.6 mg/dL).

On admission, the patient was hypovolemic and presented with severe hyponatremia and multiple electrolyte disorders. As is often the case, volume resuscitation with normal saline resulted in brisk hypotonic diuresis and a rapid rise in serum sodium concentration. This phenomenon has been frequently reported in beer potomania [5,8]. Given the low serum osmolality (225 mOsm/kg), the ADH should have been suppressed, resulting in limited osmolar clearance. To mitigate this condition, we promptly switched to 5% dextrose as free water and administered desmopressin (DDAVP) and vasopressin [5]. Nevertheless, the serum sodium increased significantly on day one, exceeding the recommended threshold of 10-12 mmoL/L within 24 hours [5,8,9].

Hypokalemia is also a common and clinically significant complication in beer potomania. In our case, the potassium level was critically low (1.3 mmol/L) and refractory to initial correction. Although multiple causes of hypokalemia have been reported, including inadequate intake, chronic diarrhea, cirrhosis-enhanced aldosterone activity, and increased urine flow rate to the distal nephron, hypomagnesemia may also contribute to renal loss of potassium, which is known to impair renal potassium reabsorption [7,9]. In our case, serum potassium improved only after the correction of hypomagnesemia. Notably, initial urine studies supported renal potassium loss; a urinary potassium-to-creatinine ratio (K/Cre) of 24.8 mmol/g and a transtubular potassium gradient (TTKG) of 8.2, indicating inappropriate potassium excretion despite severe hypokalemia. Moreover, sustained hypokalemia causes metabolic alkalosis, and the resultant elevation in serum pH facilitates the efflux of intracellular H ions and potassium influx into the cell. This mechanism may also contribute to the sustained hypokalemia in our case, which is associated with severe metabolic alkalosis (bicarbonate, 45.2 mmol/L; pH, 7.55). Although metabolic alkalosis itself is unlikely to induce vasopressin resistance, it may have amplified intracellular potassium shifts, further reducing serum potassium concentration and exacerbating ADH unresponsiveness.

**Figure 3:**
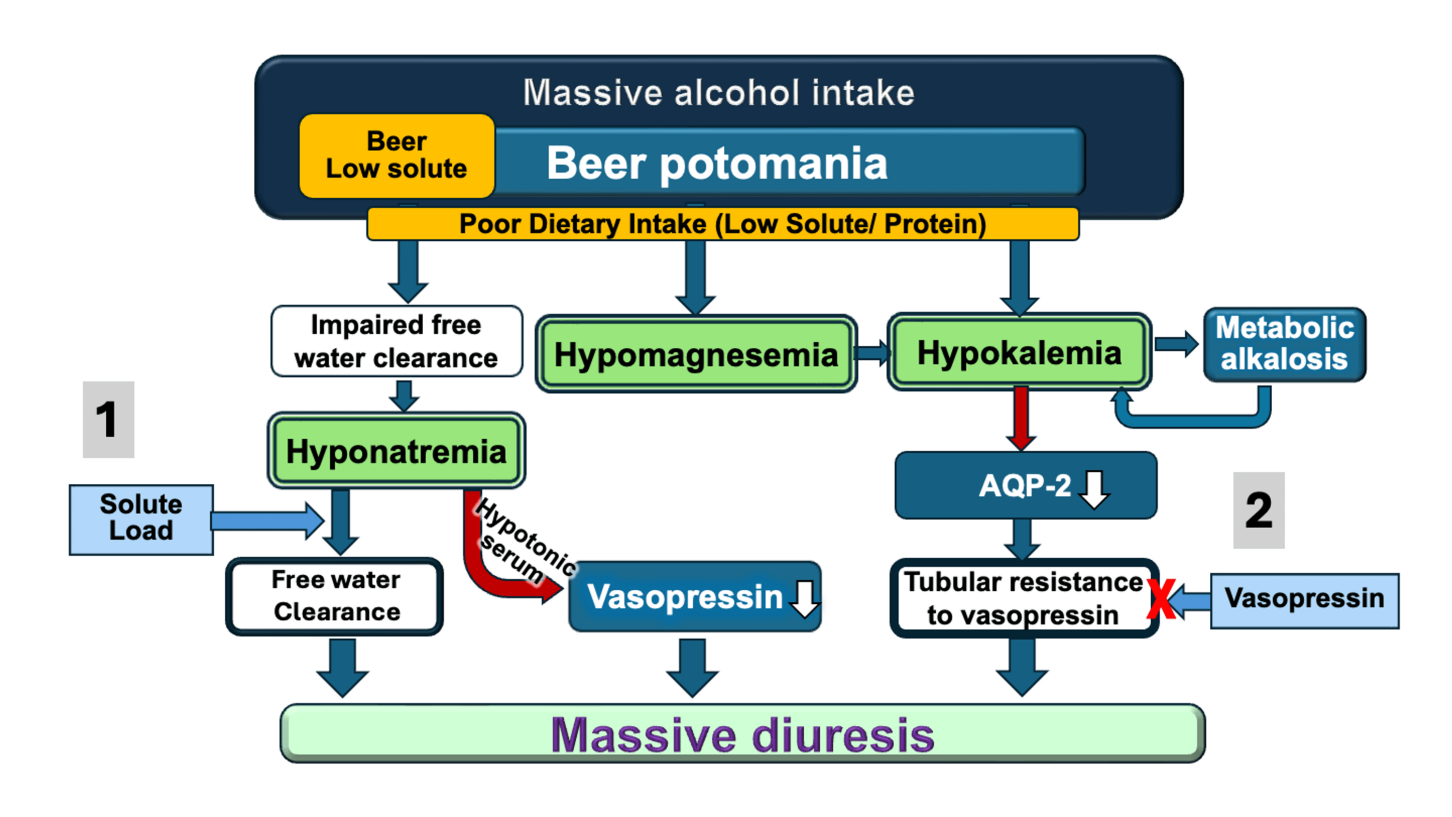
Schematic diagram illustrating the relationship between electrolyte disorders and massive diuresis in this case of beer potomania. AQP-2: aquaporin-2. Excessive beer consumption combined with low dietary solute intake resulted in impaired free water clearance and hypotonic hyponatremia. Upon ICU admission, a solute load triggered massive diuresis (1), and despite vasopressin administration, polyuria persisted due to tubular resistance likely caused by severe hypokalemia and hypomagnesemia (2). Gradual electrolyte correction restored renal responsiveness, leading to the resolution of polyuria. The figure was constructed based on our current experience, with reference to the conventional concept of beer potomania and chronic alcohol-use disorder [1,2].

Although the initial urine osmolality (143 mOsm/kg) may suggest some ADH effect, this was still inappropriately low in the condition of marked hyponatremia and polyuria. Persistent polyuria was observed despite vasopressin administration on day one (Figure 2). This suggested a transient vasopressin-resistant state, possibly due to hypokalemia-induced downregulation of the aquaporin-2 (AQP2) channel in the renal collecting ducts. Sustained hypokalemia is known to cause renal histopathological changes, including tubular vacuolation and interstitial fibrosis [10]. From the aspect of renal pathophysiology, hypokalemia is reported to be associated with nephrogenic diabetes insipidus resistant to ADH [11]. Moreover, hypokalemia has been shown to cause autophagic degradation of AQP2 channels, leading to impaired water reabsorption even in the presence of ADH and inducing downregulation of this channel expression [12,13]. In our case, the gradual decrease in urine volume occurred only after serum potassium levels exceeded 2.0 mmol/L, supporting the hypothesis that correcting hypokalemia restored AQP2 responsiveness to ADH. Although direct causality cannot be confirmed, similar mechanisms have been demonstrated in experimental data in animals and humans [12-14]. The pathophysiological sequence of events in this case is summarized schematically in Figure 3.

Notably, the interruption of vasopressin infusion at 0.03 units/min on day two resulted in a recurrence of polyuria and a sharp increase in sodium (19 mmol/L in 24 hours). This may reflect that endogenous ADH secretion was still suppressed due to hypo-osmolality, hyponatremia (121 mmol/L), and a low BUN (2.7 mg/dL), and the renal tubular response to ADH was partially restored. In such cases, continued vasopressin may be necessary until both osmotic drive and tubular ADH responsiveness are fully recovered. Although tachyphylaxis to vasopressin has been reported in some clinical settings, our patient repeatedly responded to resumed vasopressin therapy, suggesting that renal sensitivity to ADH was preserved.

Finally, our patient had multiple risk factors for ODS, including chronic alcohol use, liver cirrhosis, malnutrition, and severe hypokalemia [15,16]. Despite early use of vasopressin and 5% dextrose, sodium correction exceeded recommended thresholds. Previous reviews report ODS in up to 18% of beer potomania cases, even with careful monitoring and management [3,16]. Fortunately, our patient did not exhibit neurological sequelae by the time of ICU discharge, possibly due to close monitoring and the early initiation of vasopressin. MRI was not performed, so subclinical ODS cannot be entirely ruled out, and continued neurological observation is warranted. Nevertheless, this case emphasizes the importance of anticipating ADH-resistance diuresis and the need for extreme caution when discontinuing vasopressin.

## Conclusions

Patients with beer potomania often develop multiple electrolyte disturbances, including severe hyponatremia due to impaired solute intake and limited free water clearance. In addition, severe hypokalemia, either alone or with hypomagnesemia, may cause renal refractoriness to ADH and sustained diuresis. These abnormalities can delay treatment response and complicate sodium correction, indicating the need for comprehensive and individualized therapeutic strategies for fluid and electrolyte management, along with close monitoring. Hormone supplementation, such as vasopressin, may be required; however, it should be discontinued carefully to prevent sodium overcorrection and subsequent osmotic demyelination. It is crucial to recognize these complex interactions to ensure safe and appropriate management in this condition.
